# Absorption and reduced scattering coefficients in epidermis and dermis from a Swedish cohort study

**DOI:** 10.1117/1.JBO.28.11.115001

**Published:** 2023-11-21

**Authors:** Hanna Jonasson, Ingemar Fredriksson, Sara Bergstrand, Carl Johan Östgren, Marcus Larsson, Tomas Strömberg

**Affiliations:** a Linköping University, Department of Biomedical Engineering, Linköping, Sweden; b Perimed AB, Järfälla, Stockholm, Sweden; c Linköping University, Department of Health, Medicine, and Caring Sciences, Linköping, Sweden; d Linköping University, Centre of Medical Image Science and Visualization Linköping, Sweden

**Keywords:** skin, optical properties, absorption, scattering, cohort study, diffuse reflectance spectroscopy

## Abstract

**Significance:**

Knowledge of optical properties is important to accurately model light propagation in tissue, but *in vivo* reference data are sparse.

**Aim:**

The aim of our study was to present *in vivo* skin optical properties from a large Swedish cohort including 3809 subjects using a three-layered skin model and spatially resolved diffuse reflectance spectroscopy (Periflux PF6000 EPOS).

**Approach:**

Diffuse reflectance spectra (475 to 850 nm) at 0.4 and 1.2 mm source–detector separations were analyzed using an inverse Monte Carlo method. The model had one epidermis layer with variable thicknesses and melanin-related absorptions and two dermis layers with varying hemoglobin concentrations and equal oxygen saturations. The reduced scattering coefficient was equal across all layers.

**Results:**

Median absorption coefficients (${\mathrm{mm}}^{-1}$) in the upper dermis ranged from 0.094 at 475 nm to 0.0048 at 850 nm and similarly in the lower dermis from 0.059 to 0.0035. The reduced scattering coefficient (${\mathrm{mm}}^{-1}$) ranged from 3.22 to 1.20, and the sampling depth (mm) ranged from 0.23 to 0.38 (0.4 mm separation) and from 0.49 to 0.68 (1.2 mm separation). There were differences in optical properties across sex, age groups, and BMI categories.

**Conclusions:**

Reference material for skin optical properties is presented.

## 1 Introduction

Knowing the optical properties of tissue is of central importance in accurately modeling light propagation.^1^ These optical properties describe photon scattering and absorption in tissue and can be used to simulate the amount of backscattered light from an illuminated tissue surface using diffusion theory or the Monte Carlo technique. In *in vivo* bio-optical applications, it is common to design instruments that make use of inverse modeling for estimating these properties from spatially, temporarily, and/or spectrally resolved diffusely backscattered light intensities. One such instrument is the PeriFlux 6000 EPOS (enhanced perfusion and oxygen saturation) system in which inverse Monte Carlo is used to analyze spatially and spectrally resolved data to estimate parameters of direct clinical values including hemoglobin tissue fraction and oxygen saturation.^2^^–^^4^

The tissue model used in the EPOS system is a three-layer skin model with one epidermal layer and two dermal layers. The importance of using a multilayer tissue model accounting for epidermal pigmentation has recently been emphasized by Phan et al.,^5^ who used spatial frequency domain imaging (SFDI) and observed a decrease in the intersubject coefficient of variation of reduced scattering, with increasing wavelengths coinciding with a lower melanin absorption coefficient; they proposed that the variation in scattering at shorter wavelengths was largely due to the inability of the semi-infinite homogeneous light transport model to adequately extract optical properties in subjects with darker skin. Wang et al.^6^ also used a three-layer skin model with two epidermal layers and found that the melanin absorption coefficient times the layer thickness could be more accurately assessed compared with assessing melanin absorption alone. Furthermore, it was not necessary to know the epidermal thickness using independent measurement with, e.g., optical coherence tomography or microscopy.

It is not always necessary to individually estimate a full set of optical properties. This includes many therapeutic optical techniques and some simplified diagnostic optical techniques that only target, e.g., hemoglobin oxygen saturation estimations.^7^ In these cases, tabulated data on optical properties can be used to better predict treatment outcomes and design analysis algorithms. Tabulated data for a wide range of tissue types have been previously presented.^1^^,^^8^^–^^10^ The applied techniques and sample sizes differ between these studies. Tabulated data are not always consistent between studies, and the origin of these differences is unclear. Data may come from widely different tissue samples, such as *in vivo* or *ex vivo* samples. The aim of this study is to present *in vivo* forearm skin tissue optical properties from a large Swedish cohort using a three-layered skin model and a probe-based diffuse reflectance spectroscopy (DRS) system with short source–detector fiber separations. Data are given for reduced scattering to complement our previous study on a subset of subjects.^11^ In addition, epidermal absorption and dermis absorption are given in the visible wavelength range. We also present the corresponding sampling depth for each of the two source–detector separations.

## 2 Method

### 2.1 Instrumentation

**Fig. 1:**
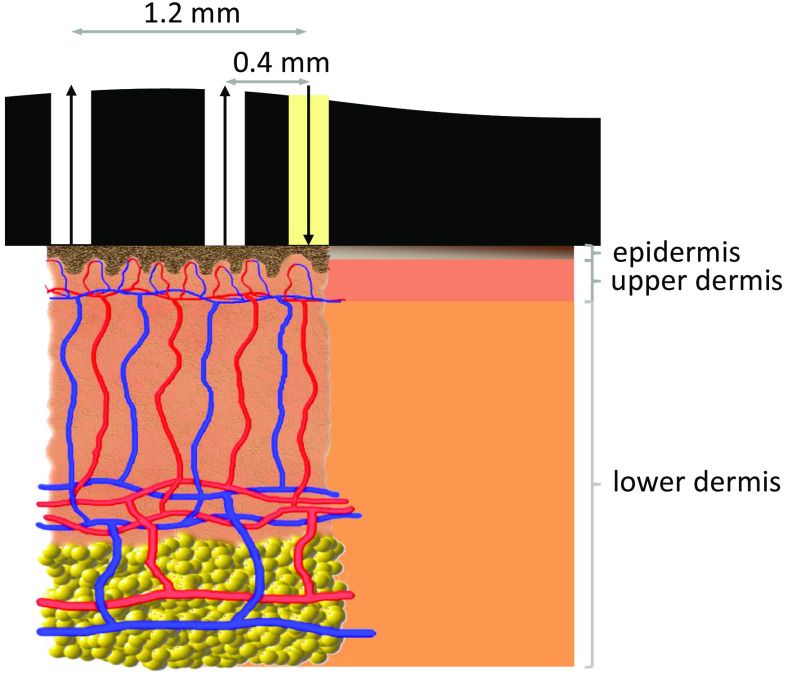
Skin anatomy illustration with epidermis, dermis, and subcutis layers and capillary loops in papillary dermis; upper and lower dermal vascular plexus and subcutaneous vessels; and vertical feeding arterioles and venules (lower left). The probe with a DRS source fiber and two detector fibers at separations 0.4 and 1.2 mm from the source (double arrow heads). The three-layer skin model with epidermis drawn 75  μm thick (fitting parameter), upper dermis 0.2 mm thick, and infinite lower dermis (curly brackets; here drawn as 1.75 mm thick; lower right).

In this study, we used a Periflux 6000 EPOS system (Perimed AB, Järfälla, Stockholm, Sweden). The system consists of a PF6011 laser Doppler unit with a laser light source at 785 nm, a spectroscopy unit with two spectrometers (AvaSpec-ULS2048L, Avantes BV, the Netherlands), a broadband white light source (Avalight-HAL-S, Avantes BV), and a fiber-optic probe. Only data from the spectroscopy unit were used to determine the absorption and scattering properties in this study. In the fiber-optic probe, two detecting fibers were placed at separations of 0.4 and 1.2 mm from the emitting fiber (see Fig. 1). The spectrometers attached to those fibers utilized the 475 to 850 nm wavelength range in the inverse Monte Carlo analysis, except data in the 770 to 810 nm range, where an optical notch filter suppressed the laser light.

### 2.2 Three-Layered Skin Model

To analyze the diffuse reflectance spectra, a three-layered skin model was used. The model has previously been described in detail.^2^^,^^12^ Briefly, the model consisted of one epidermis layer with variable thickness and two dermis layers. The upper dermis had a fixed thickness of 0.2 mm, roughly corresponding to the upper vascular plexus of the dermis, and the lower was assigned an infinite thickness. Deeper vascular plexus as well as subcutis fat have a minor effect on the reflected spectra and are therefore not modeled separately.

The reduced scattering coefficient (${\mathrm{mm}}^{-1}$) with three adaptable parameters was modeled to be equal across the layers and was described by Jacques as^1^

$$\mu_{s}^{\prime }(\lambda )=\alpha ((1-\gamma ){\left(\frac{\lambda }{600}\right)}^{-\beta }+\gamma {\left(\frac{\lambda }{600}\right)}^{-4}),$$ (1)

where α equals $\mu_{s}^{\prime }$ at 600 nm, β is the Mie scattering decay, and γ describes the fraction of Rayleigh scattering.

Melanin absorption (${\mathrm{mm}}^{-1}$) is described according to Ref. 1 as

$$\mu_{a,\mathrm{mel}}(\lambda )=48.4{\left(\frac{\lambda }{550}\right)}^{-\beta_{\mathrm{mel}}},$$ (2)

where $\beta_{\mathrm{mel}}$ is an adaptable parameter allowing the model to fit various types of melanin. Multiplying Eq. (2) with the melanin content of the epidermis layer ($f_{\mathrm{mel}}$) provides the absorption coefficient $\mu_{a,\mathrm{epi}}$ of the top epidermis layer.

The absorption coefficient in dermis layer n is calculated as

$$\mu_{a,n}(\lambda )=f_{\text{blood},n}c_{\mathrm{vp},n}(s_{n}\mu_{a,\mathrm{oxy}}(\lambda )+(1-s_{n})\mu_{a,\text{deoxy}}(\lambda )),$$ (3)

where $f_{\text{blood}}$ is the fraction of blood, $c_{\mathrm{vp}}$ is a factor compensating for the vessel packaging effect,^13^^,^^14^ and s is the hemoglobin oxygen saturation. The blood was modeled to have a hematocrit of 43% and a mean cell hemoglobin concentration of 345  g/L RBC. Values for $\mu_{a,\mathrm{oxy}}$ were based on Zijlstra et al.,^15^ and values for $\mu_{a,\text{deoxy}}$ were based on Prahl,^16^ as these correlate best with data from other sources^17^^–^^19^ and in our experience offer the best model fit.^3^^,^^20^

Modeled spectra were calculated by adding the effect of absorption, using Beer–Lambert’s law, in each layer to Monte Carlo-simulated models generated at distinct levels of epidermis thicknesses and scattering, where the total pathlength in each layer was stored for each photon. Two-dimensional interpolation was utilized for epidermis thicknesses and scattering between the simulated levels. The modeled spectra were fitted to measured diffuse reflectance spectra (between 475 and 850 nm) at 0.4 and 1.2 mm source–detector separation using a nonlinear search algorithm. Multiple starting points in the parameter space were utilized to assure the global optimal solution. When the measured spectra were similar to the previous spectra, the previous solution was used as the starting point for a new time point.

The model has been validated previously using simulations of light transport and tissue-mimicking optical phantoms,^2^^–^^4^ with known optical and geometrical properties.

The sampling depth was assessed from Monte Carlo-simulated data for the optical properties valid at each wavelength for both source–detector separations. To quantify the sampling depth for each simulation, a point cloud was generated for random positions from all photon paths of all detected photons. The same number of points was used for all detected photons, regardless of their total path length. The points were then weighted with the final weight of the detected photon. The cumulative weight of all points was calculated as a function of the depth. The sampling depth was defined as the depth where the cumulative sum reached 63% ($1-e^{-1}$) of the total sum.

### 2.3 In Vivo Data

Measurements were performed on the volar forearm of 3809 subjects, aged 50 to 65 years, the majority with Caucasian skin types. All subjects were recruited within a large multicenter study in Sweden, the Swedish CArdioPulmonary bioImage Study (SCAPIS).^21^ SCAPIS has been approved as a multicenter trial by the Ethics Committee at Umeå University (Dnr 2010-228-31M with amendment, EPN Umeå) and adheres to the Declaration of Helsinki. The diffuse reflectance measurements and the analysis of data have been approved by the ethics committee in Linköping (Dnr 2018/156-31). Written informed consent was obtained from all subjects.

The subjects were asked to refrain from large meals and coffee for 3 h, nicotine for 4 h, and alcohol for 12 h prior to the measurements. The subjects were also asked to omit medications the morning of the study; except for anticoagulants, contraceptives, or medications for Parkinsons disease, diabetes, epilepsy, chronic pain, and/or spasticity. The subjects were acclimatized in a temperature-controlled room and rested in a supine position for 15 min before the start of the measurements. The fiber-optic probe was attached to the right forearm using double-sided adhesive tape, avoiding visible veins, pigmented nevi, and hair. The full protocol included a 5-min baseline, 5-min arterial occlusion of the forearm using a blood pressure cuff rapidly inflated to above systolic pressure (250 mmHg), and a 10-min reperfusion after release of the pressure cuff. Only data from the baseline measurements are included in this study. All parameters are calculated as the mean over the first 3 min of the baseline measurement.

### 2.4 Statistical Analysis

The characteristics of the study population were described as mean ± standard deviation (SD) or as proportions for categorical variables. Due to skewed distributions, the absorption, the scattering coefficient, the scattering parameters, and the sampling depth were described as medians (interquartile range IQR; 25th to 75th percentiles). We tested differences in the absorption and scattering parameters between males and females using the nonparametric Mann–Whitney U-test, and across age groups and BMI groups, respectively, using the nonparametric Kruskal–Wallis test (nonparametric ANOVA). Dunn’s *post hoc* test with Bonferroni correction was used for pairwise comparisons. A p-value<0.05 was regarded as significant.

## 3 Results

Subjects were excluded from the analysis due to missing data files (n=28), aborted measurement upon request by the subject (n=3), data acquisition failure (n=16), data quality issues [large model fit error^2^ or uncertain model parameters due to low amount of blood^22^ (n=113)], and failure of the subjects to follow the given instructions regarding coffee and medication intake prior to the measurement occasion (n=123). In total, 283 subjects were excluded leaving 3526 subjects included in the analysis. Characteristics of the included subjects are available in Table 1.

**Table 1:** Characteristics of the included subjects.

		N=3526
	Age (years)	57.5 ± 4.4
	Females	1726 (49.0%)
	BMI ($\mathrm{kg}/m^{2}$)	26.9 ± 4.4
	Systolic blood pressure (mmHg)	133 ± 18
	Diastolic blood pressure (mmHg)	84 ± 10
Medical history	Diabetes	259 (7.3%)
	Hypertension	705 (20.0%)
Smoking status	Current	337 (9.6%)
	Never	1979 (56.1%)

Values are mean (±SD) or n (%). BMI, body mass index.

**Fig. 2:**
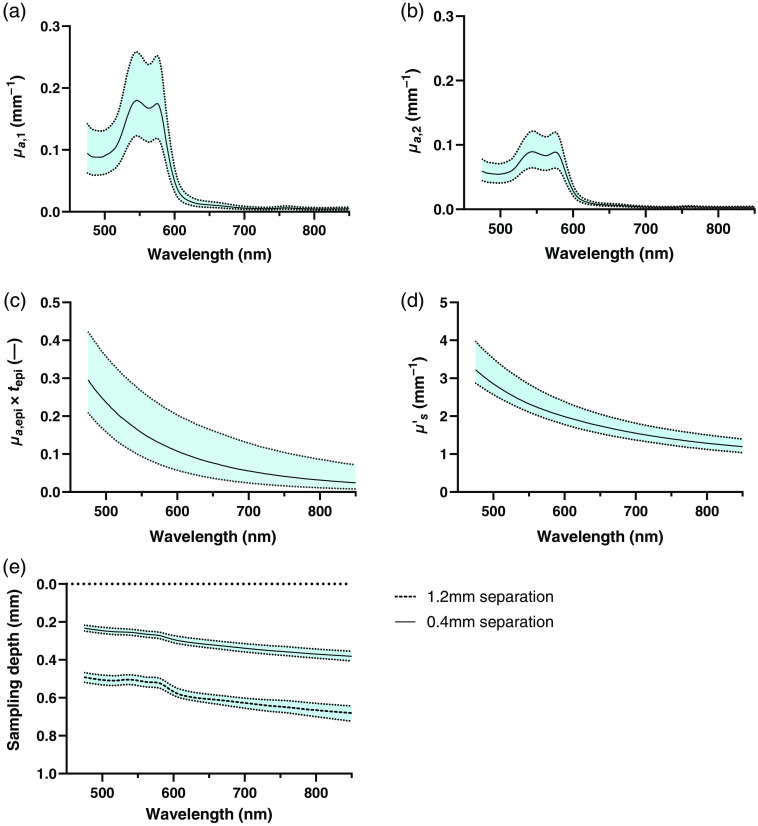
Absorption coefficient in the (a) upper dermis layer and (b) lower dermis layer. (c) Total amount of melanin absorption (absorption coefficient in the epidermis times epidermis thickness, $\mu_{a,\mathrm{epi}}\times t_{\mathrm{epi}}$). (d) Reduced scattering coefficient. (e) Sampling depth for the two source–detector separations (0.4 and 1.2 mm). Data are given as medians (and 25th to 75th percentiles).

The median absorption and reduced scattering coefficients in the wavelength range 475 to 850 nm, and their normal variation (25th to 75th percentiles) are shown in Fig. 2. Figures 2(a) and 2(b) present the absorption coefficient in the upper ($\mu_{a,1}$) and lower dermis ($\mu_{a,2}$), respectively, and Fig. 2(c) presents the total amount of melanin absorption in the epidermis [absorption coefficient in epidermis ($\mu_{a,\mathrm{epi}}$) times epidermis thickness ($t_{\mathrm{epi}}$)]. Figure 2(d) presents the reduced scattering coefficient ($\mu_{s}^{\prime }$), and Fig. 2(e) presents the sampling depth.

The absorption coefficient in the two dermis layers, the total amount of melanin absorption in the epidermis [absorption coefficient in epidermis ($\mu_{a,\mathrm{epi}}$) times epidermis thickness ($t_{\mathrm{epi}}$)], the reduced scattering coefficient, and the sampling depth for the two source–detector separations (0.4 and 1.2 mm) for selected wavelengths are presented in Table 2.

**Table 2:** Dermal absorption coefficients, total amount of melanin absorption, reduced scattering coefficient, and sampling depth for the two source–detector separations (0.4 and 1.2 mm).

Wavelength (nm)	$\mu_{a,1}$ (${\mathrm{mm}}^{-1}$)	$\mu_{a,2}$ (${\mathrm{mm}}^{-1}$)	$\mu_{a,\mathrm{epi}}\times t_{\mathrm{epi}}$ (—)	$\mu_{s}^{\prime }$ (${\mathrm{mm}}^{-1}$)	Sampling depth 0.4 mm separation (mm)	Sampling depth 1.2 mm separation (mm)
475	0.094 (0.063 to 0.14)	0.059 (0.045 to 0.078)	0.30 (0.21 to 0.42)	3.22 (2.87 to 3.97)	0.23 (0.22 to 0.25)	0.49 (0.47 to 0.52)
500	0.091 (0.061 to 0.13)	0.055 (0.041 to 0.071)	0.24 (0.16 to 0.36)	2.85 (2.57 to 3.52)	0.25 (0.23 to 0.26)	0.51 (0.48 to 0.53)
525	0.13 (0.087 to 0.19)	0.071 (0.053 to 0.094)	0.19 (0.12 to 0.31)	2.56 (2.32 to 3.14)	0.25 (0.24 to 0.27)	0.51 (0.48 to 0.53)
550	0.18 (0.12 to 0.25)	0.088 (0.063 to 0.12)	0.16 (0.094 to 0.27)	2.32 (2.11 to 2.84)	0.26 (0.24 to 0.28)	0.51 (0.48 to 0.54)
575	0.18 (0.12 to 0.25)	0.089 (0.064 to 0.12)	0.13 (0.073 to 0.23)	2.14 (1.93 to 2.59)	0.27 (0.25 to 0.29)	0.52 (0.49 to 0.55)
600	0.049 (0.032 to 0.071)	0.029 (0.021 to 0.038)	0.11 (0.057 to 0.20)	1.99 (1.78 to 2.38)	0.29 (0.27 to 0.31)	0.57 (0.54 to 0.59)
625	0.016 (0.010 to 0.023)	0.0094 (0.0069 to 0.013)	0.090 (0.046 to 0.18)	1.86 (1.65 to 2.20)	0.31 (0.29 to 0.33)	0.60 (0.57 to 0.61)
650	0.011 (0.0071 to 0.017)	0.0066 (0.0048 to 0.0089)	0.077 (0.036 to 0.16)	1.74 (1.54 to 2.05)	0.32 (0.30 to 0.34)	0.61 (0.58 to 0.63)
675	0.0085 (0.0054 to 0.013)	0.0051 (0.0037 to 0.0069)	0.065 (0.030 to 0.14)	1.64 (1.45 to 1.93)	0.33 (0.31 to 0.35)	0.62 (0.59 to 0.64)
700	0.0060 (0.0039 to 0.0089)	0.0037 (0.0027 to 0.0049)	0.056 (0.024 to 0.13)	1.55 (1.37 to 1.82)	0.34 (0.32 to 0.36)	0.63 (0.60 to 0.66)
725	0.0050 (0.0033 to 0.0073)	0.0031 (0.0023 to 0.0041)	0.048 (0.20 to 0.12)	1.47 (1.30 to 1.72)	0.35 (0.32 to 0.37)	0.64 (0.61 to 0.67)
750	0.0060 (0.0040 to 0.0088)	0.0038 (0.0029 to 0.0049)	0.041 (0.016 to 0.10)	1.40 (1.24 to 1.64)	0.36 (0.33 to 0.38)	0.65 (0.61 to 0.68)
775	0.0055 (0.0037 to 0.0080)	0.0036 (0.0028 to 0.0047)	0.036 (0.013 to 0.095)	1.34 (1.17 to 1.57)	0.36 (0.34 to 0.38)	0.66 (0.62 to 0.69)
800	0.0048 (0.0032 to 0.0070)	0.0033 (0.0025 to 0.0042)	0.032 (0.011 to 0.086)	1.29 (1.12 to 1.51)	0.37 (0.34 to 0.39)	0.67 (0.63 to 0.70)
825	0.0045 (0.0030 to 0.0069)	0.0033 (0.0025 to 0.0043)	0.028 (0.0094 to 0.078)	1.24 (1.08 to 1.45)	0.38 (0.35 to 0.40)	0.67 (0.64 to 0.71)
850	0.0048 (0.0032 to 0.0073)	0.0035 (0.0027 to 0.0046)	0.024 (0.0079 to 0.072)	1.20 (1.04 to 1.40)	0.38 (0.36 to 0.41)	0.68 (0.64 to 0.72)

Data are given as medians (25% to 75% IQR).

The estimated median epidermal thickness was 0.063 mm (IQR: 0.013 to 0.191), the fraction of melanin ($f_{\mathrm{mel}}$) was 0.050 (IQR: 0.016 to 0.21), and the $\beta_{\mathrm{mel}}$ was 4.3 (IQR: 2.9 to 5.8). The median blood tissue fraction $f_{\mathrm{blood}}$ in the upper dermis layer was 0.011 (IQR: 0.0071 to 0.016) and in the lower dermis was 0.0075 (IQR: 0.0058 to 0.010). The median oxygen saturation (equal in the two dermis layers) was 50% (IQR: 41 to 60).

There was a significant difference in absorption coefficient at 570 nm in both layers across sex, with males having higher absorption coefficients compared with females (Table 3). There was also a significant difference in scattering parameters, with the scattering parameters α and β being higher for males compared with females and the scattering parameter γ being lower. No difference in the total amount of melanin absorption was observed.

**Table 3:** Absorption coefficients, total amount of melanin absorption, and scattering parameters for males and females.

	All	Males	Females	p value
n	3526	1800	1726	
$\mu_{a,1}$ at 570 nm (${\mathrm{mm}}^{-1}$)	0.17 (0.12 to 0.25)	0.18 (0.12 to 0.26)	0.16 (0.11 to 0.24)	<0.001
$\mu_{a,2}$ at 570 nm (${\mathrm{mm}}^{-1}$)	0.086 (0.062 to 0.12)	0.097 (0.073 to 0.13)	0.075 (0.054 to 0.10)	<0.001
$\mu_{a,\mathrm{epi}}*t_{\mathrm{epi}}$ at 570 nm (—)	0.13 (0.077 to 0.24)	0.14 (0.077 to 0.24)	0.13 (0.076 to 0.23)	n.s.
α (${\mathrm{mm}}^{-1}$)	1.99 (1.78 to 2.38)	2.04 (1.84 to 2.44)	1.94 (1.75 to 2.30)	<0.001
β (—)	0.82 (0.55 to 1.2	0.89 (0.60 to 1.24)	0.75 (0.50 to 1.04)	<0.001
γ (—)	0.31 (0.21 to 0.39)	0.29 (0.14 to 0.37)	0.33 (0.24 to 0.41)	<0.001

Data are given as medians (25 to 75% IQR). p values refer to comparisons between males and females using the Mann–Whitney U-test.

**Fig. 3:**
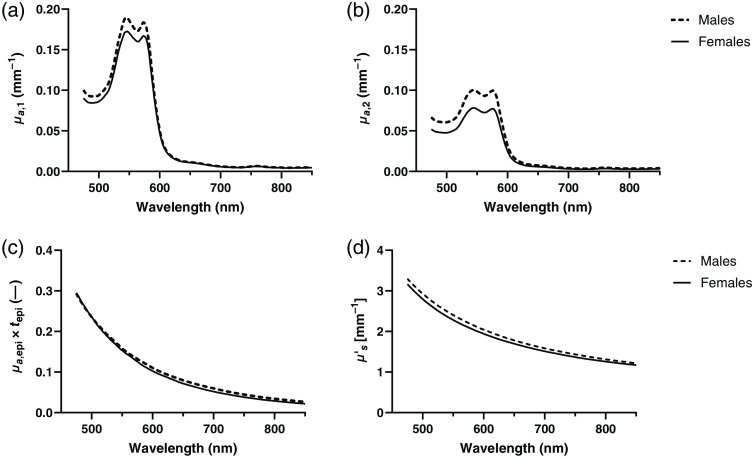
Absorption coefficient in the (a) upper dermis layer and (b) the lower dermis layer. (c) Total amount of melanin absorption (absorption coefficient in the epidermis times epidermis thickness, $\mu_{a,\mathrm{epi}}\times t_{\mathrm{epi}}$). (d) Reduced scattering coefficient. Median values for males (dashed) and females (solid).

Figure 3 shows the median absorption and reduced scattering coefficients in the wavelength range 475 to 850 nm for males (dashed) and females (solid), separately. Figures 3(a) and 3(b) present the absorption coefficient in the upper ($\mu_{a,1}$) and lower dermis ($\mu_{a,2}$), respectively. Figure 3(c) presents the total amount of melanin absorption in the epidermis [absorption coefficient in epidermis ($\mu_{a,\mathrm{epi}}$) times epidermis thickness ($t_{\mathrm{epi}}$)], and Fig. 3(d) presents the reduced scattering coefficient ($\mu_{s}^{\prime }$).

The analysis showed a statistically significant difference in the scattering parameter α across the different age groups (p<0.001, Table 4). *Post hoc* analysis demonstrated that the youngest age group displayed a significantly higher scattering parameter α compared with both older age groups (p<0.05). However, no significant differences were observed in any other scattering parameters, in the absorption coefficients, or in the total amount of melanin absorption.

**Table 4:** Absorption coefficients, total amount of melanin absorption, and scattering parameters for different age groups.

	Age			
	50.1 to 54.9	55.0 to 59.9	60.0 to 65.3	p value
n	1199	1136	1191	
Females, n (%)	594 (49.5)	557 (49.0)	575 (48.3)	
$\mu_{a,1}$ at 570 nm (${\mathrm{mm}}^{-1}$)	0.17 (0.11 to 0.24)	0.17 (0.12 to 0.25)	0.18 (0.12 to 0.25)	n.s.
$\mu_{a,2}$ at 570 nm (${\mathrm{mm}}^{-1}$)	0.086 (0.064 to 0.12)	0.086 (0.062 to 0.11)	0.087 (0.061 to 0.12)	n.s.
$\mu_{a,\mathrm{epi}}\times t_{\mathrm{epi}}$ at 570 nm (—)	0.13 (0.076 to 0.24)	0.13 (0.078 to 0.23)	0.14 (0.076 to 0.25)	n.s.
α (${\mathrm{mm}}^{-1}$)	2.04 (1.83 to 2.42)^a^	1.98 (1.78 to 2.35)	1.96 (1.74 to 2.33)	<0.001
β (—)	0.84 (0.57 to 1.18)	0.81 (0.55 to 1.15)	0.81 (0.54 to 1.15)	n.s.
γ (—)	0.31 (0.21 to 0.40)	0.31 (0.22 to 0.38)	0.31 (0.21 to 0.40)	n.s.

Data are given as medians (25% to 75% IQR).

p values refer to comparisons across age groups using the Kruskal–Wallis test.

a Significant difference between youngest age group and both older age groups p<0.05 using Dunn’s *post hoc* test with Bonferroni correction.

A linear regression analysis was conducted to examine the relationship between age and the scattering parameter α. The model was significant (p<0.001) with a regression beta of −0.009.

We found statistically significant differences in both absorption coefficients and total amount of melanin absorption between BMI categories (underweight, healthy weight, overweight, and obese) (p<0.001). *Post hoc* analysis showed that the absorption coefficients in both dermis layers were significantly higher in overweight and obese (p<0.05) compared with healthy weight subjects. The absorption coefficient in the upper dermis layer was also significantly higher for obese compared with overweight individuals. Additionally, the total amount of melanin absorption was significantly lower in overweight and obese individuals compared with healthy weight individuals and was also significantly lower for obese compared with overweight individuals (Table 5).

**Table 5:** Absorption coefficients, total amount of melanin absorption, and scattering parameters for different BMI categories.

	BMI				
	<18.5	18.5 to 24.9	25.0 to 29.9	≥30.0	p value
	Underweight	Healthy weight	Overweight	Obese	
n	13	1233	1549	731	
Females, n (%)	11 (84.6)	718 (58.2)	647 (41.8)	350 (47.9)	
$\mu_{a,1}$ at 570 nm (${\mathrm{mm}}^{-1}$)	0.14 (0.12 to 0.19)	0.16 (0.11 to 0.22)^a^	0.17 (0.12 to 0.25)^b^	0.19 (0.14 to 0.28)	<0.001
$\mu_{a,2}$ at 570 nm (${\mathrm{mm}}^{-1}$)	0.059 (0.052 to 0.089)	0.080 (0.056 to 0.11)^a^	0.089 (0.065 to 0.12)	0.093 (0.067 to 0.12)	<0.001
$\mu_{a,\mathrm{epi}}\times t_{\mathrm{epi}}$ at 570 nm (—)	0.13 (0.069 to 0.31)	0.15 (0.085 to 0.25)^a^	0.14 (0.075 to 0.23)^b^	0.12 (0.065 to 0.21)	<0.001
α (${\mathrm{mm}}^{-1}$)	2.01 (1.83 to 2.71)	1.97 (1.75 to 2.39)	2.00 (1.80 to 2.41)	1.98 (1.79 to 2.30)	n.s.
β (—)	0.57 (0.38 to 1.17)	0.80 (0.53 to 1.17)	0.82 (0.56 to 1.15)	0.86 (0.59 to 1.18)	n.s.
γ (—)	0.35 (0.20 to 0.47)	0.32 (0.22 to 0.40)	0.31 (0.22 to 0.39)	0.30 (0.21 to 0.38)	n.s.

Data are given as medians (25% to 75% IQR). p values refer to comparison across BMI groups using the Kruskal–Wallis test.

a Significant difference between healthy weight and both overweight and obese.

b Significant difference between overweight and obese, p<0.05, using Dunn’s *post hoc* test with Bonferroni correction.

**Fig. 4:**
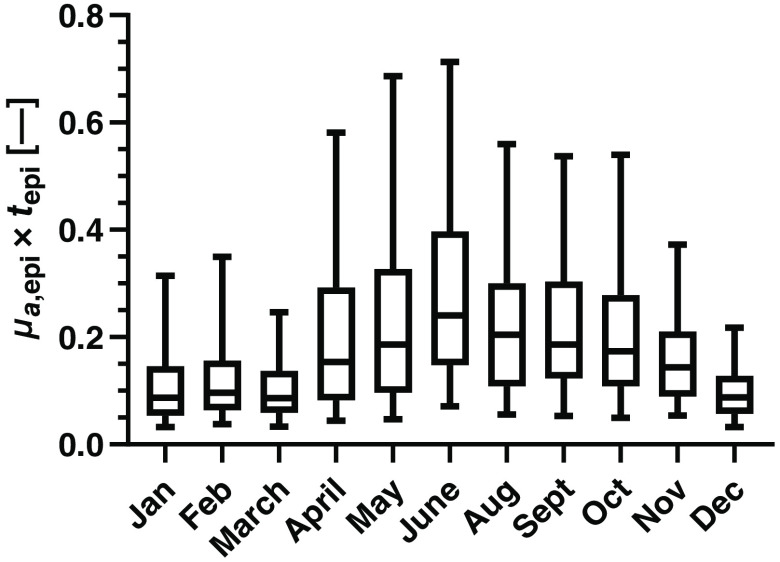
Epidermis melanin absorption (absorption coefficient in epidermis times epidermis thickness, $\mu_{a,\mathrm{epi}}\times t_{\mathrm{epi}}$) at 570 nm over the year.

There was a difference in the epidermal melanin absorption, e.g., at 570 nm, over the year (Fig. 4). No measurements were conducted during July due to the summer holiday. The average epidermal melanin absorption ranged from 0.086 in March to 0.24 in June at 570 nm. The difference between the winter season (December to February) of 0.091 (IQR: 0.058 to 0.14) and the summer season (June to September) or 0.21 (IQR: 0.13 to 0.33) was significant (p<0.001).

## 4 Discussion

Tabulated values on the absorption coefficient of skin tissue obviously depend on the skin type and skin site that are being examined. There is also a dependency on what measurement technique is used: both the hardware design and the inverse algorithm may affect the estimated optical properties. Often the layered structure of skin tissue is treated as a single homogeneous layer in the inverse algorithm, resulting in only a single-compound absorption value being reported. These values are therefore a weighted average of all included skin layers, with the weights depending on the sampling depth. Single-layer models have also been found to not fully describe the diffusely backscattered spectra from skin tissue, which can affect the error when used in an inverse algorithm to estimate optical properties.^23^ This type of estimation error and the dependency on the sampling depth become less of a problem when using inverse algorithms based on multilayer models, in which each layer can be assigned a unique absorption property. For multilayered models, the sampling depth instead gives an indication of how well each layer was sampled. In this study, the average sampling depth varied with wavelength and was 0.23 to 0.38 mm for the short fiber separation and 0.49 to 0.68 mm for the long fiber separation. This indicates that the deepest layer, starting at an average depth of 0.26 mm, was properly sampled to enable a separate estimation of $\mu_{a}$ for each layer. When using inverse modeling, the applied tissue model needs to be complex enough, with a sufficient degree of free parameters, to fully explain the measured spectra. In this study, the misfit between measured and modeled spectra was generally very low, which supports that our model complexity is sufficient and that no major chromophore with unique characteristics is missing.

The EPOS system has been validated using both theoretical light transport simulations and physical models. Light transport simulations with varying tissue model properties showed that the hemoglobin oxygen saturation s was estimated within 4% and the tissue fraction of blood $f_{\text{blood}}$ within ∼20%.^2^ Validations with measurements on liquid tissue-mimicking optical phantoms during deoxygenation of blood using yeast yielded s estimations within 5% and $f_{\text{blood}}$ within 11%.^3^ That study also showed that the accuracy of $\mu_{s}^{\prime }$ was within 15% using two-layered silicone phantoms including ${\mathrm{TiO}}_{2}$ as a scattering agent.^3^ Errors below 20% for the dermal absorption properties and reduced scattering show that these parameters can be estimated with sufficient accuracy for clinical needs. Hence, when comparing data between studies, differences are either true physiological differences or due to differences in how data were analyzed or the probing depth for the applied techniques. One exception with EPOS is the epidermal thickness, which has a poor accuracy.^3^ The epidermal absorbance $\mu_{a,\mathrm{epi}}t_{\mathrm{epi}}$ may have a better accuracy.^6^

The sampling depth is a property that is not only wavelength dependent but also directly affected by the used fiber separation in DRS, or similarly, the spatial-frequencies in spatial-frequency-domain techniques. In the 540 to 580 nm range, where blood is the dominant chromophore for pale skin, the relative influence from superficial melanin on the backscattered light intensity is suppressed. In this spectral range, Zonios et al.^24^ reported data that, converted as described by Lister et al.,^10^ resulted in a $\mu_{a}$ of 0.27 to $0.30\;{\mathrm{mm}}^{-1}$ at the volar side of the forearm in subjects with skin type III. These are average values that include probe measurements from both normal skin and nevi. Kono and Yamada^25^ also reported an average $\mu_{a}$ of about $0.3\;{\mathrm{mm}}^{-1}$ measured at the volar side of the forearm in 198 subjects originating from Japan using a spatial-frequency-domain imaging technique. Both Kono and Zonios displayed a higher $\mu_{a}$ compared with the $\mu_{a}$ of $0.18\;{\mathrm{mm}}^{-1}$ found in our top dermal layer. This difference is most likely explained by their approximation of skin tissue to be a single homogeneous layer including both blood and melanin, whereas our multilayer approach allows melanin to only influence absorption in our epidermal layer and not in our top dermal layer.

The absorption coefficient at 570 nm, a factor that is strongly dependent on the amount of blood in tissue, was found to increase significantly with BMI in both dermal layers. This is in line with previously presented trends of increased skin redness with BMI.^26^ The absorption coefficient at 570 nm was also higher in males than females, a relationship that has been reported before.^25^ We found no significant correlation with age, which might be due to only including subjects in the fairly narrow 50 to 65-year age range.

We observed a median $f_{\text{blood}}$ of 1.1% in the upper dermis layer and 0.75% in the lower dermis layer. The estimated lower fraction in the second dermis layer is likely an effect of our DRS setup, with a maximal fiber separation of 1.2 mm and a sampling depth of up to 0.68 mm. This limited sampling depth mainly includes superficial capillaries, whereas deeper blood-rich and well-saturated arterial vascular plexus have a significantly smaller impact on the estimated fraction in the lower dermis. This assumption is also supported by the close-to-zero oxygen saturation detected after a 5-min occlusion provocation, indicating that the sampled volume mainly consists of capillary vessels where oxygen is allowed to diffuse into the surrounding tissue.^27^

Our $f_{\text{blood}}$ values are lower than the 2.2% measured by Yudovsky et al.^28^ using SFDI and a two-layered Monte Carlo model in which only the bottom layer contained blood. This difference could be explained by Yudovsky’s setup, in which spatial frequencies up to $0.25\;{\mathrm{mm}}^{-1}$ and near infrared light (650 to 1100 nm) were used, which most likely resulted in a significantly greater sampling depth. Tsui et al.^29^ estimated an $f_{\text{blood}}$ value of around 0.2% in skin tissue (ventral arm) using a DRS system with a maximal fiber separation of 0.73 mm, a spectral range of 410 to 760 nm, and a three-layered Monte Carlo model in which only the bottom layer contained blood. The significantly lower $f_{\mathrm{blood}}$ values presented by Tsui et al. can be explained by their poor model fit in the 500 to 600 nm wavelength region where the amount of hemoglobin is expected to significantly impact the amount of backscattered light.

Changes in epidermal thickness and in melanin concentration have a similar effect on the diffuse reflectance spectra; i.e., increasing melanin concentration has a comparable effect as increasing the thickness of the epidermis. Therefore, there is a large degree of uncertainty when evaluating those parameters individually. However, the product of the two, which reflects the total amount of melanin in the epidermis, can be estimated with greater accuracy in the inverse Monte Carlo process. In Table 2, we, therefore, present this product rather than presenting the parameters separately. By normalizing this product with the average estimated epidermal thickness, one can obtain the average absorption coefficient of the epidermal layer. This coefficient is directly influenced by the tissue fraction of melanosomes. By assuming that the absolute level of melanosome absorption is given by the expression presented by Jacques et al.,^30^ i.e., $\mu_{a}(\lambda )=1.7\times 10^{12}\lambda^{-3.48}$ (${\mathrm{cm}}^{-1}$), we obtain an average melanosome tissue fraction of 3.9%. This fraction is just above the 1% to 3% interval for light-skinned Caucasians given by Jacques^31^ and just below the 5% level for Fitzpatrick skin type I presented by Saager et al.,^32^ a level that appears reasonable for a primarily fair-skinned Swedish cohort. The estimated fractions above rely on an accurate estimation of epidermal thickness. This is difficult on an individual level, as discussed above, but on a population-average level, it is more likely that our 63  μm estimated thickness is accurate. This is supported by the findings of Sandby-Møller et al.,^33^ who presented an epidermal thickness of 75  μm for the dorsal side of the arm and by Lee and Hwang,^34^ who presented an epidermal thickness of 74  μm for the volar forearm.

The total amount of melanin was found to significantly depend on BMI, with the highest amount being found in the healthy weight range (BMI 18.5 to 24.9). The total amount of absorption due to melanin was also found to vary over the year ranging from 0.086 in March to 0.24 in June (median). This is expected, as the Swedish climate typically calls for Sun exposure of the forearms during summer starting from late spring (April to May). The total amount of melanin in skin was found not to depend on age or sex.

We observe a median reduced scattering coefficient of $1.99\;{\mathrm{mm}}^{-1}$ at 600 nm (i.e., α in our reduced scattering model). This is slightly larger compared with the average $1.75\;{\mathrm{mm}}^{-1}$ (compiled from graph) reported by Phan et al.^35^ based on four subjects with Fitzpatrick skin types I to II. This difference is most likely explained by the low number of subjects, the slightly different measurement locations (dorsal forearm), and the methodological differences, with Phan et al. using SFDI with a maximal spatial frequency of $0.2\;{\mathrm{mm}}^{-1}$. This frequency range indicates a larger sampling depth compared with our DRS probe, for which the maximal fiber separation is 1.2 mm. Kono and Yamada^25^ measured the scattering coefficient on the volar side of the forearm on 198 subjects originating from Japan using a spatial-frequency-domain imaging technique. They reported on an average scattering coefficient of $11.9\;{\mathrm{mm}}^{-1}$ at 600 nm using a combination of two different Henyey–Greenstein phase functions in their inverse Monte Carlo algorithm. Their complex phase function had an estimated anisotropy factor of g=0.67, resulting in an average reduced scattering coefficient of $3.9\;{\mathrm{mm}}^{-1}$, a value that is about twice as high as what is reported in this study as well as by Phan et al.^35^ and others.^24^^,^^36^ Whether this significant difference originates from differences in study populations or methodological approaches is unknown.

Our results show that the reduced scattering coefficient decreases significantly with age and is lower for females than males. Kono and Yamada^25^ found a similar relationship with age but an inverse relationship with sex. We found no correlation between the reduced scattering coefficient and BMI, in contrast to the negative correlation found by Rodriguez et al.^37^ This could be due to Rodriguez et al. studying a different skin tissue site (inner wrist) in a more BMI-diverse population.

## 5 Conclusion

In this study, *in vivo* values for skin absorption and reduced scattering properties were presented from a cohort of 3809 subjects. The presented values on absorption and reduced scattering were coherent with many previous values. However, tabulated values on absorption and reduced scattering coefficients obviously depend on what skin type and skin site were being examined. There was also a dependency on what measurement technique was used: both the hardware design and the inverse algorithm may affect the estimated optical properties. Our results also showed differences in optical properties across sex, age group, BMI category, and expected sun exposure according to the season of the year.

## Data Availability

The data that support the findings of this study are available from the Swedish CArdioPulmonary bioImage Study (SCAPIS) Data Access Board. Restrictions apply to the availability of these data, which were used under license for the current study and so are not publicly available. However, the data are available from the authors upon reasonable request and with permission of the SCAPIS National Steering Committee.
